# Leaky gut biomarkers in casein- and gluten-rich diet fed rat model of autism

**DOI:** 10.1515/tnsci-2020-0207

**Published:** 2021-12-31

**Authors:** Hussain Al Dera, Bahauddeen Alrafaei, Muneerah I. AL Tamimi, Hanan A. Alfawaz, Ramesa Shafi Bhat, Dina A. Soliman, Sameera Abuaish, Afaf El-Ansary

**Affiliations:** Basic Medical Science Department, College of Medicine, King Saud bin Abdulaziz University for Health Sciences, Riyadh, Saudi Arabia; King Abdullah International Medical Research Center (Kaimrc), Riyadh, Saudi Arabia; Home Economic Department, Prince Sattam Bin Abdulaziz University, Al-Kharj, Saudi Arabia; Department of Food Science and Nutrition, King Saud University, Riyadh, Saudi Arabia; Department of Biochemistry, College of Science, King Saud University, Riyadh, Saudi Arabia; Botany and Microbiology Department, College of Science, King Saud University, Riyadh, Saudi Arabia; Department of Basic Sciences, College of Medicine, Princess Nourah Bint Abdulrahman University, P.O. Box 84428, Riyadh 11671, Saudi Arabia; Central Research Laboratory, Female Center for Medical Studies and Scientific Section, King Saud University, P.O. Box 22452, Riyadh, Saudi Arabia

**Keywords:** autism, leaky gut, zonulin, lipid peroxides, glutathione, gut microbiota

## Abstract

It is proposed that gluten- and casein-rich diets (GRD and CRD) can synergistically exacerbate dysbiosis as comorbidity in autism by worsening leaky gut that affects the brain through the gut–brain axis. In this study, 35 young male rats were divided into 7 groups, Group 1 serves as control; Group 2, clindamycin (CL)-treated; and Group 3, propionic acid (PPA)-induced rodent model of autism. These three groups were fed standard diet until the end of the experiment. Groups 4–7 are rats treated similarly with CL and PPA, then fed on CRD or GRD until the end of the experiment. Serum zonulin, glutathione (GSH), lipid peroxides, and gut microbial composition were measured in the seven studied groups. Data demonstrate the significant increase in serum zonulin as marker of leaky gut in the CL-treated groups fed on CRD or GRD. Lipid peroxides were significantly higher in the serum of GRD-fed rats compared to CRD-fed or normal diet-fed rats. GSH was much lower in CL-treated groups fed on CRD or GRD compared to PPA-treated rats fed on both diets. Both diets differentially affected the diversity of the gut microbiota. This study demonstrates that CRD and GRD exacerbates leaky gut, according to serum zonulin, which was used as marker for increased gut permeability.

## 1 Introduction

Globally, about 20% of children and adolescents demonstrate mental, behavioral, and neurodevelopmental disorders and these are the prominent reasons of disability in young individuals [1,2]. The etiology of most intellectual disorders, including neurodevelopmental disorders such as autism spectrum disorders (ASD), is unknown, but genetic influences, biochemical abnormalities, and environmental stressors are contributed in the etiology [3]. The interplay between the brain, leaky gut, and the gut microbiota has become a rapidly growing area of research. Increased leaky gut or intestinal permeability has been examined with regard to emotional, behavioral, and neurodevelopmental disorders such as ASD [4,5].

Children with ASD have been found to display an increased immune reactivity against proteins such as gliadin (a gluten-specific protein) and casein (a protein in dairy products). It was demonstrated that children with ASD have high rates of antibodies against gliadin and casein (i.e., anti-gliadin and anti-casein) [6]. Interestingly they also have antibodies against dipeptidyl peptidase 4 (DPP4) as digestive enzyme which is involved both in digestion and in regulation processes such as immune function, pain perception, intracellular signal transduction coupled to control of cell migration and proliferation [7]. It is very important in the processing of gliadin. It is well known that gliadin is broken down into several peptides among which are gliadinomorphin-7 (GM7) [8] an immune reactive peptide with “opioid activity”, and thus it stimulates opioid receptors in the body [9]. Further degradation of GM7 is therefore necessary and its cleavage is catalyzed by DPP4 which is not very active in ASD patients [10]. As Vojdani et al. reported, the presence of anti-DPP4 would theoretically diminish the amount of circulating DPP4, with concomitant increase in GM7 and the probability of downstream, opioid-like response [10]. It should be highlighted that casein and other dietary peptides are similarly degraded to intermediary substances with opioid properties (e.g., casomorphin) [11] and together these potentially harmful peptides have been termed “exorphins” [12]

Zonulin is a family of architecturally related peptides that are well-known as physiologic modulator of intestinal tight junctions [13]. Zonulin appears to be the primary modulator that is involved in the regulation of gut–blood and blood–brain barriers permeability that were recently related to the pathology of ASD [14]. Zonulin is also a potential inflammatory marker and contributes to intestinal innate immunity. Increased IP coinciding with inflammation has been described in mental disorders in children [15].

1. Zonulin has been associated with low-grade inflammation and autoimmune diseases, as well as ASD, which might have an autoimmune component [16]. Furthermore, in ASD, higher serum zonulin has been associated with social impairment compared to controls [4,5].
2. It is well known that oxidative stress plays a key role in the early phase of intestinal injury, and it implements as the activating factor for intestinal barrier dysfunction, thus prompting the immune imbalance and inflammation [17]. ASD patients with gastrointestinal (GI)–comorbidity and celiac disease (CD) patients demonstrate oxidative stress which is known to worsen in case of gluten-rich diets (GRD). Much lower glutathione (GSH) and higher lipid peroxides are previously recorded in blood of these patients [18,19,20].

Propionic acid (PPA) as enteric fatty acid bacterial fermentation metabolite has the ability to induce extensive effects on gut, brain, and behavior. Brain tissue from PPA-treated rats demonstrates numerous neurochemical alterations for instance, neuroinflammation, glutamate excitotoxicity, oxidative stress, GSH depletion, and altered membrane phospholipid consistent with findings in ASD patients. Moreover, PPA has additional bioactive properties on neurotransmitter systems, mitochondrial function, intestinal permeability, and immune response. All these PPA-induced alterations are consistent with the signs and the suggested principal etiological mechanisms of ASD and thus, support the use of PPA in rats as a valid animal model of ASD [21,22]. Most recently, our team tested the application of two interventional treatments, Bifidobacterium probiotic treatment and fecal transplantation as two strategies to treat dysbiosis, and social interaction impairment in oral PPA-administered rats. Both treatments were effective in modulating the overgrowth of gut Clostridium bacteria and social impairment in PPA-rodent model of autism [23].

Additionally, transplanting *Clostridium difficile-*rich gut microbiota of ASD patients into pregnant mice is sufficient to promote the autism-like behavior in offspring [24]. These findings highlight the contribution of the gut–brain axis in the etiology of autism and recommend possible interventions in a preclinical model of autism.

A fundamental hypothesis proposed that antibiotic treatment kills native intestinal bacterial inhabitants that usually compete with pathogenic bacteria such as *C. difficile*. It is well known that a single dose of clindamycin (CL) significantly reduces the diversity of the intestinal microbiota for at least 28 days, and induces a remarkable increase in *C. difficile* as PPA producers. Thus, CL treatment could be used as indirect strategy to test the neurotoxic effect of PPA through the induction of *C. difficile* overgrowth [25].

This information initiates our interest to measure serum zonulin as marker of impaired gut microbiota together with GSH and lipid peroxides as antioxidant and oxidative stress status markers, respectively, in CL-treated and PPA-induced rodent model of autism fed on standard diet (SD), casein-rich diet (CRD), and GRD. This might help in understanding and ascertain the relationship between casein and gluten sensitivity, oxidative stress, and leaky gut in ASD.

## 2 Material and methods

### 2.1 Formulation of CRDs and GRDs

Both CRD and GRD were formulated by Dyets for laboratory animal’s research and all constituents are shown in Tables 1 and 2, respectively.

**Table 1:** Ingredients of CRD

Ingredient	kcal/g	g/kg	kcal/kg
Casein, high nitrogen	3.58	200	716
l-Cystine	4	3	12
Sucrose	4	100	400
Cornstarch	3.6	397.486	1430.9496
Dyetrose	3.8	132	501.6
Soybean oil	9	70	630
*t*-Butylhydroquinone	0	0.014	0
Cellulose	0	50	0
Mineral mix #210025	0.88	35	30.8
Vitamin mix # 310025	3.87	10	38.7
Choline bitartrate	0	2.5	0
Total		1,000	3760.0496

**Table 2:** Ingredients of GRD

Ingredient	kcal/g	g/kg	kcal/kg
Wheat gluten	3.68	200	920
Sucrose	4	100	400
Cornstarch	3.6	342.786	1234.0296
Dyetrose	3.8	132	501.6
Soybean oil	9	70	630
*t*-Butylhydroquinone	0	0.014	0
Cellulose	0	50	0
Mineral mix #210025	0.88	35	30.8
Vitamin mix # 310025	3.87	10	38.7
Choline bitartrate	0	2.5	0
l-Methionine	4	3	12.00
l-Lysine	4	3.7	14.80
l-Threonine	4	1	4.00
Total		1000.00	3785.9296

### 2.2 Experimental animal model

Total of 35 healthy male rats weighing 80–100 g were obtained from Prince Naif Animal Research Centre and used in all studies. All experimental procedures for evaluating ASD development were performed on 3 week-old animals. The rats were randomly divided into either control or CL-treated and PPA-treated (ASD) groups. Animals were allowed access to standard rodent chow and tap water *ad libitum*.

**Figure 1:**
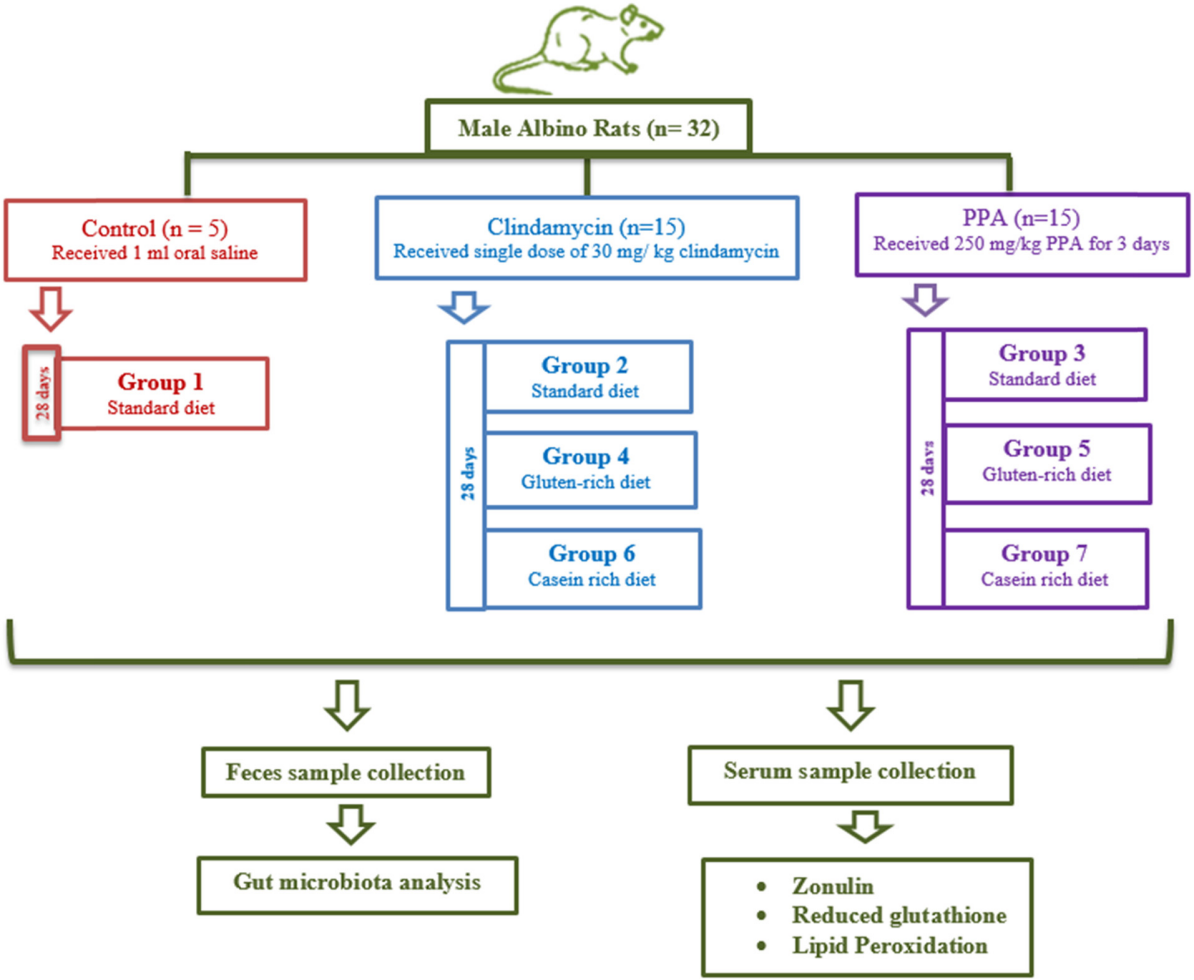
Illustration of the experimental design.


All rats were housed 2 per cage under controlled environmental conditions (22 ± 1°C) and an established light:dark photoperiod (12:12 h; lights on: 07:00). The experimental procedure is illustrated in Figure 1. On the first day of testing, rats were designated to receive either 1 mL of oral saline (control *n* = 5), or single dose of 30 mg/kg CL (*n* = 15) or an oral dose of 250 mg/kg PPA (*n* = 15) dissolved in distilled water for 3 days [26]. Later, the CL- and PPA-treated groups were sub-divided to feed on either SD (*n* = 5), GRD (*n* = 5), or CRD (*n* = 5) for 28 days.

**Ethical approval:** The research related to animals’ use has been complied with all the relevant national regulations and institutional policies for the care and use of animals. All animal study procedures were approved by the Ethics Committee for Animal Care and Use of King Saud University (No. KSU-SE-19-54).

### 2.3 Collection of serum

Blood sample was collected by direct cardiac puncture in a plane tube without anticoagulant. Serum was collected after centrifugation of blood at 1,100 × *g* for 10 min. The collected serum samples were immediately stored at −80°C until use. ELISA and biochemical assays were performed in the BMS lab at COM, KASU-HS.

#### 2.3.1 Measurement of lipid peroxidation

The extent of lipid peroxidation was determined by measuring the levels of the lipid peroxidation products and thiobarbituric acid (TBA) reactive substances, mainly malondialdehyde. According to the TBA test by Ruiz-Larrea et al. [27], boiling the samples with TBA at a low pH results in the development of a pink chromogen that can be measured at 532 nm.

#### 2.3.2 Measurement of reduced GSH

Reduced GSH was measured using the GSH assay, carried out using tissue homogenate according to the method of Beutler and Kelly [28].

#### 2.3.3 Measurement of serum zonulin

Serum zonulin in all groups were measured using ELISA kit, a product of MyBioSource. In this assay, the plate has been pre-coated with rat zonulin antibody. Samples are added to different wells, and serum zonulin binds to antibodies coated on the wells. This is followed by the addition of biotinylated rat zonulin antibody which binds to zonulin in the serum sample. Then, Streptavidin-HRP is added and binds to the biotinylated zonulin antibody. After incubation, unbound Streptavidin-HRP is washed away during the washing step. Substrate solution is then added and color develops relative to the amount of serum rat zonulin. The reaction was ended by adding acidic solution and read at 450 nm. 0.1–40 ng/mL, and sensitivity of 0.042 ng/mL.

### 2.4 Gut microbiota analyses from fecal samples

Fecal samples of all animal groups were collected and stored at −80°C. Each sample was homogenized using a sonicator for 30 s in 0.1 M pH 7.2 PBS (at a 1:10 weight/volume ratio). The solutions were centrifuged at 3000 rpm for 5 min at 4°C. One milliliter of the fecal supernatant was serially diluted in 9 mL of sterile PBS solution 4 times [29]. Bacterial populations were estimated by growth on nutrient agar, MacConkey agar, 5% sheep blood agar, and Mueller–Hinton agar, while yeast populations were enumerated on Sabouraud dextrose agar using 100 µL of sample from each animal separately. All culture media were incubated at 37°C under aerobic conditions for 18–24 h. The experiment was repeated twice. The average number of bacteria/yeast per plate was recorded. Gram staining and biochemical tests were used to identify the bacterial strains [30].

### 2.5 Statistical analysis

SPSS version 16.0 was used for data analysis; the results were expressed as mean value ± SE. The data were checked for normality using the Shapiro-Wilk test. Not more than one outlier was excluded to achieve normality for some of the measures. Extreme outliers were determined using box plots and removed when they were more than 3× the data’s interquartile range. Serum biochemical assays were done by two-way ANOVA followed by a Tukey’s *post hoc* analysis for multiple comparisons with differences considered significant at *P* ≤ 0.05.

## 3 Results and discussion

Recent studies suggest potential, but unconfirmed, associations between dietary, metabolic, infective, and GI influences and the behavioral improvements or exacerbations of ASDs. PPA as a metabolic end product of multiple ASD-associated bacteria, such as *Desulfovibrio, Clostridia*, and *Bacteroidetes*, is reasonably linked to ASDs and can induce widespread effects on gut, brain, and behavior [23].

The main findings of the present translational study is a trend of an increase (*P* = 0.09) in serum zonulin in PPA-autism model fed on ND, CRD, and GRD compared to control healthy model fed on ND. Significant increase in serum zonulin was recorded in CL-autism model fed on GRD compared either to control healthy model fed on ND (*P* ≤ 0.0001), PPA-autism model fed on both CRD and GRD (*P* ≤ 0.05), or CL-autism model fed on ND (*P* ≤ 0.01). On the other hand, CRD demonstrates significant increase in serum zonulin compared to control healthy and CL-autism model fed on ND.

In an attempt to understand the mechanisms behind the elevation of zonulin as marker of leaky gut in CRD- and GRD-fed rats, we can highlight that both casein as the major milk protein and gliadin as wheat gluten-derived protein are hydrolyzed resulting in the release of heptapeptide hβ-casomorphin-7 (hBCM7), bβ-casomorphin-7 (bBCM7), and α-gliadin yields GM7. Different amino acids at positions 3–5 of these proteins together with the presence of 2–3 proline residues give them unique conformations which are resistant to the action of intestinal proteolytic enzymes [31]. Each of these homologous proline-rich peptides has the ability to initiate opioid receptors [32,33,34].

**Figure 2:**
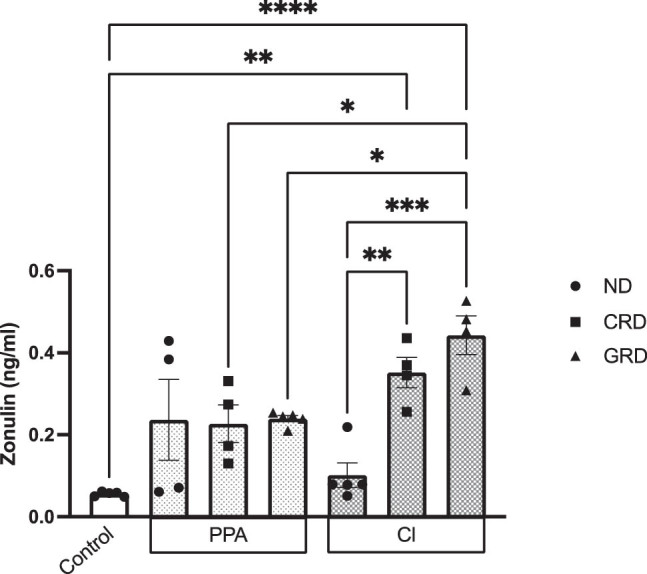
Zonulin levels in the plasma of PPA- and CL-treated animals consuming ND, CRD, or GRD. Data presented are mean values ± standard error. Significant one-way ANOVA was followed by multiple comparisons using Tukey’s *post hoc* test ****P ≤ 0.0001, ***P ≤ 0.001, **P ≤ 0.01, and *P ≤ 0.05.

PPA-treated animals fed on ND, CRD, and GRD might indicate higher permeability with a *P*-value of 0.09, but a higher sample size is recommended to be confident to suggest this. The significant increase in serum zonulin in CRD- and GRD-CL-treated rats compared to CL-treated rats fed on ND could help to suggest that both GRD-and CRD worsen intestinal hyper permeability as realistic consequences of feeding both diets to CL-treated rats but not in PPA-treated rats. Significant levels of zonulin were observed as marker of intestinal permeability in GRD- and CRD-fed CL-treated rats compared to those fed on ND (*P* ˂ 0.001 and 0.01, respectively) (Figure 2). This could be related to the opioid-excess hypothesis of autism. It is well documented that children with ASD have shown impaired protein digestion together with intestinal hyper permeability, and raised levels of urinary peptides of dietary origin classified as exorphins (exogenous opioids) among which are casomorphins, and gliadinomorphins as breakdown products of casein and gluten [35]. A1 β-casein involves both opioid and non-opioid signaling pathways, it increases total GI transit time and colonic myeloperoxidase as opioid receptor-mediated effects, and DPP-4 activity modulation as opioid receptors – independent together with the pro-inflammatory effects [36]. Gliadin as wheat-derived exorphine stimulates the production of pro-inflammatory cytokines for instance interleukin (IL)-1β, tumor necrosis factor-α, IL-6,-8,-15, and induces the release of zonulin as marker of leaky gut [37,38,39]. Zonulin activates epidermal growth factor receptor which in turn increases intestinal permeability through the change in tight junction proteins [40,41].

Based on the excess opioid theory, casomorphins and gliadinomorphins agonist of opioid receptors can induce systemic effects and are able to cross the blood–brain barrier [35]. Treatment of these ASD subjects with an extended gluten-free, casein-free diet for 2–4 years resulted in significant decrease in urinary peptide levels as well as satisfactory improvement in the autistic behavioral measures [35,42,43], regardless of abundant intake of meat and fish protein, which can help to relate CRD and GRD to excess opioid theory [35]. More support can be found in considering multiple animal studies which demonstrated that inhibition of gut peptidases, specifically DPP-4, results in increased levels of urinary peptides of dietary origin [10,43,44].

**Figure 3:**
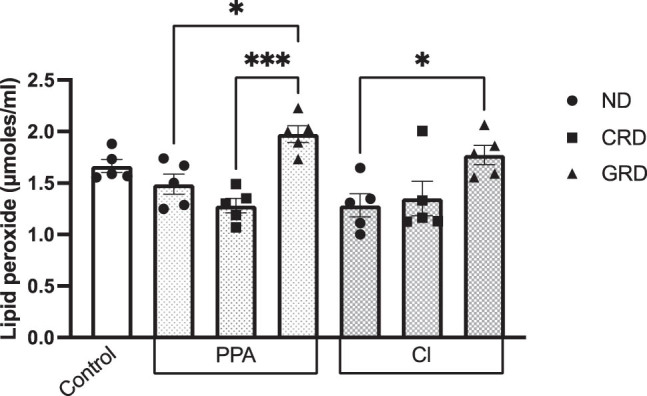
Lipid peroxide levels in the PPA- and CL-treated animals consuming ND, CRD, or GRD. Data presented are mean values ± standard error. Significant one-way ANOVA was followed by multiple comparisons using Tukey’s *post hoc* test ***P ≤ 0.001, **P ≤ 0.01, and *P ≤ 0.05.

**Figure 4:**
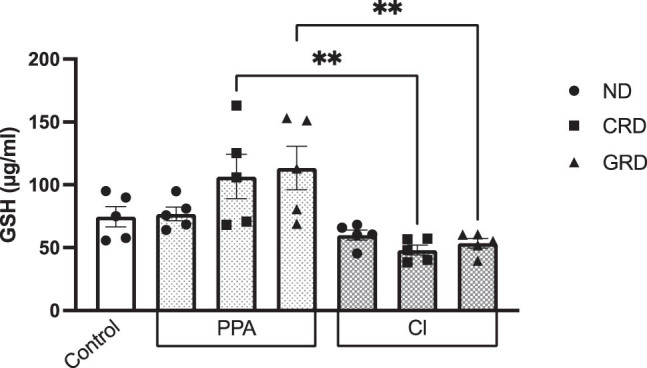
GSH levels in the PPA- and CL-treated animals consuming either ND, CRD, or GRD. Data presented are mean values ± standard error. Significant one-way ANOVA was followed by multiple comparisons using Tukey’s *post hoc* test **P ≤ 0.01.

Both lipid peroxidation and GSH was unaffected in the serum of PPA- and CL-treated rats fed on ND (Figures 3 and 4). This is in contrast to the effects of both treatments on brain tissues of treated animals which demonstrate increase in lipid peroxides and GSH depletion as oxidative stress markers [26,45]. This could be explained on the basis that rat brain is especially susceptible to oxidative stress as neurotoxic effects of PPA and CL [46,47].

Lipid peroxides were significantly higher in PPA-treated rats fed on GRD compared to those fed on ND or even CRD. Similarly, increase was observed in GRD fed rats treated with CL in comparison to those on ND. This is in good agreement with numerous studies which emphasized the immunologic or molecular mechanisms of gluten toxicity, specifically demonstrating that gliadin as proline-rich peptide shows an important role in exerting cytotoxic and immunomodulatory activities, as well as triggering oxidative stress in patients with GI morbidity such as celiac disease (CD) patients [48,49,50]. It is very interesting to note that enzymatically prepared wheat gluten hydrolysate (WGH) had antioxidant effects [51,52]. The contrasting effects seen between gluten/gliadin and WGH ascertain the contribution of the incomplete digestion of gluten and its toxic effect through gliadin.

The GI tract has been related to autism through the gut–brain axis. Several reports demonstrate the overrepresentation of functional and pathological gut conditions in individuals with autism [53,54], the influence of different dietary interventions for autism [55], and the novel triad involving GI immune function–intestinal barrier permeability–gut microbiota [56] potentially being relevant to some of the autistic features in humans and rodent models. Recently in 2020, Forsyth [57] reported the decrease in the abundance of certain bacteria among which is *Moraxella* in ASD individuals compared to typically developing children. Table 3 demonstrates the remarkable decrease in *Moraxella* in CL-treated rats fed on ND as rodent model of autism (Group 2), absence in CL-treated rats fed on GRD, PPA-treated rats fed on CRD or GRD (Groups 4, 6, and 7), and a remarkable increase in CL-treated rats fed on CRD. This can find support in the recent work of Forsyth et al. [58] in which he recorded 31.9% lower abundance of *Moraxella* in individuals with ASD compared to healthy controls. Moraxella has a tendency to secrete multiple hydrolytic enzymes among which are proteases which catalysis the hydrolysis of casein that might be related to the production of casomorphins as exorphins [59].

**Table 3:** Approximate microorganism colony count/plate in all treatment groups

Isolated organisms	Media and incubation conditions	Group 1 control	Group 2 CL-ND	Group 3 CL-CRD	Group 4 CL-GRD	Group 5 PPA-ND	Group 6 PPA-CRD	Group 7 PPA-GRD
*Staphylococcus* and/or bacilli (gram-positive cocci/rods or gram-negative rods)	NA, aerobic: 37°C, 24 h	**++++**	**+++**	**+**	**+**	**+++**	**+++**	**+**
Enterobacteriaceae (gram-negative rods, lactose fermenters)	MCA, aerobic: 37°C, 24 h	**+**	**++**	**++**	**−**	**−**	**++**	**+++**
Gram-positive/gram-negative rods and cocci	BAP, aerobic: 37°C, 24 h	**+++**	**−**	**+++**	**+++**	**+++**	**++**	**−**
*Moraxella* spp. gram-negative	MHA, aerobic: 37°C, 24 h	**++**	**+**	**++**	**−**	**++++**	**−**	**−**
*Candida albicans*	SDA, aerobic: 25°C, 48 h	**−**	**−**	**−**	**−**	**−**	**−**	**++**

− = no growth; + = rare; ++ = few; +++ = moderate; ++++ = heavy. NA, nutrient agar; MCA, MacConkey agar; BAP, 5% sheep blood agar; MHA, Mueller–Hinton agar; SDA, sabouraud dextrose agar (yeast media).

Moreover, the recorded growth of *Candida albicans in* PPA-treated GRD*-*fed rats could be supported by the work of Harnett et al. [60] which detects *Candida* sp. in 33% of CD fecal specimens as disease related to gluten sensitivity compared to 0% of the control group confirming the idea that *Candida* may act as a trigger of autoimmune responses in genetically predisposed subjects. Thus, *Candida* components might theoretically contribute to CD etiology by modifying immunogenic epitopes of gluten and resulting in immune response. The reported growth of C*. albicans* in PPA-treated rats fed on GRD could be explained on the basis that *Candida* is well-adapted for growth in the gut where inflammation may disturb the inhabitant bacterial community generating conditions that favor *Candida* growth and inflammation. The recorded growth of *Candida* in PPA group fed on GRD (Table 3) could be explained through considering the opioid-excess hypothesis of autism and the ability of gliadin to initiate opioid receptors. It was shown that morphine as opioid analgesics reduced the phagocytic and fungicidal activity of macrophages towards *C. albicans*, which could explain the noticed growth in response to GRD [61,62]. The absence of *C. albicans* growth in CL-treated group fed on GRD (Group 7) could be explained through considering the early study of Kennedy and Volz [63]. It was found that CL reduced anaerobic population levels, but not enteric bacilli or aerobes, also animals prone to mucosal association by *C. albicans*. It is suggested that the strictly anaerobic bacterial populations which predominate in the gut in CL-treated rats (*Enterobacteriaceae)* are responsible for the inhibition of *C. albicans* adhesion, colonization, and diffusion from the intestinal tract (Table 3; Groups 2, 3, and 4). This explanation could be further supported through considering the most recent work of Markey et al. [64], who reported that bacterial colonization and the *C. albicans* are more easily changed by CL treatment providing perception into the microbiota response to acute CL challenge and the effect of *C. albicans* colonization on ecological resistance.

## 4 Conclusion

In conclusion, CRD and GRD can deteriorate intestinal permeability leading to higher levels of serum zonulin as marker of leaky gut. Moreover, both diets have synergistic effects on dysbiosis as a contributing factor in the development of GI comorbidity in ASD. Thus, dietary and microbial interventions to promote a healthy microbial profile in ASD patient could be suggested.
